# Impact of the quality and diversity of reference products on creative activities in online communities

**DOI:** 10.1038/s41598-024-65124-y

**Published:** 2024-07-11

**Authors:** Keisuke Sato, Kunhao Yang, Kazuhiro Ueda

**Affiliations:** 1 Graduate School of Arts and Sciences, The University of Tokyo, Tokyo, 153-8902 Japan; 2 Graduate School of Sciences and Technology for Innovation, Yamaguchi University, Yamaguchi, 755-8611 Japan

**Keywords:** Human behaviour, Computational science

## Abstract

In creativity research, an important unresolved issue relates to identifying the kind of information an individual should be exposed to in order to be highly creative. We aimed to gain insights into this issue; we collected and statistically analyzed three datasets related to three large online communities (namely Cities: Skylines, SCP-wiki, and Archive of Our Own) engaged in mod development and novel writing to examine whether the quality and diversity of other people’s products referred to have a positive effect on product generation. Our analysis revealed the following three findings: (1) the quality diversity of reference products generated by others has the most positive impact on the quality of generated products when it is neither high nor low, (2) the content diversity of reference products generated by others has a negative impact on the quality of generated products, and (3) the quality of reference products generated by others has a negative impact on the quality of generated products when it is extremely high. We conclude by discussing the implications of the findings for creativity research.

## Introduction

Creativity is a crucial human cognitive ability; it drives the advancement of society through novel ideas or creative products, helps generate innovative solutions to problems arising in society, and enriches society by inspiring artistic expression.

The degree of individual creativity is influenced by various potential factors, such as creative abilities (e.g., divergent thinking^1,2^, creative problem-solving ability^3,4^, executive abilities^5,6^, metacognitive abilities^7,8^, knowledge^9–15^, experience^16–21^, personality^22–25^, semantic and episodic memory capacity and structure^26–28^, and cognitive characteristics (which may be linked to brain structure)^29,30^. In addition, the environment surrounding an individual can affect the achievement of creativity. Previous studies have shown that certain environmental conditions, such as wide spaces^31^, messy work environments^32^, and moderate noise^33^, can facilitate creative thinking.

Receiving and utilizing information from external sources, one environmental feature that influences creativity, is particularly important for idea generation. Specifically, ideas or creative products generated by others have been identified as important for creativity in many studies^34–37^. A classic example is brainstorming, a technique that can be used to generate ideas in a group^38–40^. This technique encourages the generation of new ideas by referring to others’ ideas and promotes knowledge sharing.

In recent years, with the improvement of digital/online environments, there has been growing attention on creative activities in online communities and online innovation platforms^41–44^. In particular, available content such as ideas or products posted by others on online platforms can support creativity by serving as sources of inspiration for creative ideas^45–51^. In the field of innovation research, online platforms that lead to innovation, such as crowdsourcing, have recently been in focus, and the relationship between idea/product generation and information presentation/reference has been investigated. For example, it has been shown that the number of products viewed on the platform^52^ as well as the originality^53,54^ of prior products and their evaluation by platform users^54^ affect idea generation.

The phenomenon described above through which receiving information from external sources enhances idea generation is known as cognitive stimulation^55^ or inspiration^56^. However, this phenomenon of receiving information may lead to a kind of “fixation”^57–60^ on idea generation. Many cases both of inspiration and fixation have been reported^56^, and it is believed that various factors affect whether referring to idea examples works effectively for idea generation. In particular, regarding the nature of the presented idea examples, research has shown that referring to diverse idea examples makes inspiration more likely^61^ and that presenting common examples with low novelty makes fixation more likely^18,45^. Thus, ideas/products generated by others can have both positive and negative effects on creativity. However, our knowledge regarding the specific aspects of ideas/products that facilitate or inhibit creativity remains limited.

Against this background, we aimed to conduct an in-depth investigation to understand how the characteristics of products generated by others affect further product generation. The characteristics that we focus on are the diversity and quality of products. Many studies have clarified the importance of diversity in creativity. Diversity of knowledge^11^, diversity of words encountered^37^, diversity of identity^62^, and the functional diversity^63–65^ of teams engaged in creative activities are among the factors reported to facilitate creativity. This is because diversity promotes the discovery of new combinations of information, which increases the likelihood of generating novel ideas^66^. Further, high-quality products can potentially inspire creative activities^67^. Research has also shown that when only creative ideas were selected from a set of idea examples and presented to participants, they tended to generate higher-quality ideas compared to when all the idea examples were presented^68^. Therefore, we predicted that the higher the quality of reference products, the higher the quality of generated products.

Based on the above discussion, we proposed the following two hypotheses to examine the associations between product reference and high-quality product generation:

1. The higher the diversity of products referred to, the higher the quality of generated products.
2. The higher the quality of products referred to, the higher the quality of generated products.

To test these hypotheses, we collected and analyzed a large amount of data on creative products from three online communities actively engaged in creative activities such as video game modding (hereafter referred to as mod development) and novel writing. Researchers^12,41–44,69^ have attempted to study creativity using Internet data; expanding on prior literature, we expected that the statistical trends behind creativity, which are highly variable among and within individuals and not apparent from a small amount of data, could be quantitatively revealed in this study.

## Results

### Overview of the datasets

To investigate the role of referring to others’ products in product generation, we used three datasets collected from specific websites: (1) Cities: Skylines mod development on Steam Community, (2) original horror sci-fi novel writing records on SCP-wiki, and (3) fanfiction novels written on Archive of Our Own. The reason for using these datasets is that they comprise active communities with a high volume of user-generated content, enable the evaluation of the quality of the published creative works, and contain a large number of published works and records of referring to others’ products (i.e., in Steam Community mods, SCP-wiki stories, and Archive of Our Own novels). Within the respective websites, the referring behavior is expressed as “Favorite” on Steam Community, “Vote” on SCP-wiki, and “Bookmark” on Archive of Our Own. For each product, we obtained a list of other people’s products that were likely to have been referred to during product generation from these reference records. We defined this as the set of products referred to (hereafter, *reference products*) to produce each product (hereafter, *generated product*).

To investigate the quality of the products, we set up quantitative metrics. For Steam Community mods, we used the number of people using the mod in the game, represented by “Current Subscribers.” For SCP-wiki novels, we used the rating by other users, represented by “Rating.” For Archive of Our Own novels, we used the total number of times users selected the heart icon for the novel, represented by “Kudos.” We defined the logarithmic scale of these values as the quality of the products (see further details in the subsection “Metrics” in the “Materials and Methods” section).

It should be noted that the values of Current Subscribers and Kudos do not directly represent the quality of the products, but rather indicate the extent to which players or readers liked those products. That said, Current Subscribers and Kudos are considered to have a strong correlation with product quality. As described below, factors that could potentially influence these values were controlled for as much as possible. Therefore, in this study, we regarded Current Subscribers and Kudos as metrics for product quality. Additionally, for Cities: Skylines, there are two types of products with different characteristics coexisting—Mods and Assets—and it is considered that for the Current Subscribers value, Mods reflect functionality while Assets reflect originality^70^. However, the number of Mods is only around one-hundredth of the number of Assets and is very small. Therefore, we can consider the Current Subscribers value to be strongly dependent on originality.

### Overview of the analysis

To investigate the relationship between the characteristics of the reference products and the quality of the generated products, we conducted a regression analysis with the variables related to the characteristics of the reference products as explanatory variables and the quality of the generated products as dependent variables. As this relationship may not be captured by a simple linear model, we adopted a polynomial regression. The degree of the polynomial was selected using the Bayesian information criterion (BIC; see further details in the subsection “Polynomial Regression Model” in the “Materials and Methods” section). The BIC values for each degree of the polynomial regression model are reported in Supplementary Information Table S2.

To control for the effects of related factors, we included several variables as control variables for each dataset. In particular, as the level of creativity is influenced by the depth^10^ and breadth^11^ of knowledge and the amount of creative activity experience^17^, we controlled for variables that are thought to reflect these factors. For Steam Community, we controlled for playtime for Cities: Skylines (reflecting the depth of knowledge about Cities: Skylines), the number of games purchased on Steam (reflecting the breadth of knowledge about games), and the number of mods developed by the creator (reflecting the amount of creative activity experience). For SCP-wiki, we followed the approach adopted in a previous study^71^ and controlled for the following four variables: the number of content revisions, the time point of the initial content publication, the number of days from the author’s first participation (i.e., submission or revision) to the present (indicating the days during which the author was observed), and the number of previous author participations (corresponding to the amount of creative activity experience). For Archive of Our Own, we controlled for the number of novels written by the author (reflecting the amount of creative activity experience) and the number of fanfiction novels posted on the same site referring to the same original work. The number of fanfiction novels posted reflects the popularity of the original work and the number of readers of fanfiction novels, with a tendency for more kudos to be given to the novel with more fanfiction novels posted. The statistical information of all variables used in the analysis and their correlations are reported in Supplementary Information Table S1 and Figures S1–S3.

### Effects of the diversity of reference products

Next, we investigated how the diversity of reference products affected the quality of generated products. Here, we focused on two types of diversity: quality and content. Quality diversity is defined as the standard deviation of the quality of all products contained in a set of reference products. This indicator is said to represent one aspect of the diversity of reference products, because the larger the value, the more variation there is in the quality of reference products; if the value of this indicator is high, both high-quality and low-quality products are included in the set of reference products. The indicator of content diversity is defined as follows: for Steam Community, we use the average information content of the tags’ distribution that succinctly represent the content of the product; for SCP-wiki, we use a quantitative indicator, calculated using the novel texts (specifically, the average distance between novels calculated using sentence-BERT^72,73^); and for Archive of Our Own, we use the average information content of the distribution of original novels (see further details in the subsection “Metrics” in the “Materials and Methods” section).

To examine the relationship between the quality or content diversity of reference products and the quality of generated products, we performed a polynomial regression analysis. For each dataset, we controlled for the necessary variables (see further details in the subsection “Overview of the Analysis”). The degree of the polynomial was determined based on the BIC.

**Figure 1:**
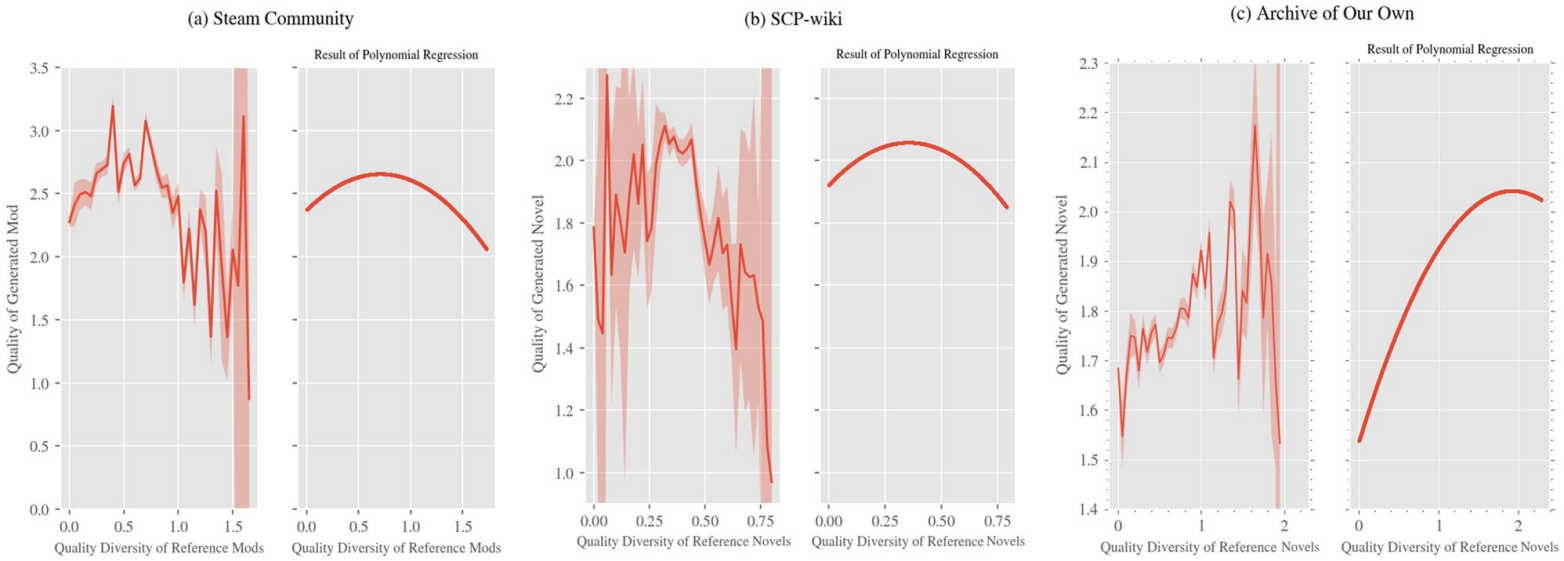
Relationship between the quality diversity of reference products and the quality of generated products. This figure shows the relationship between the standard deviation of the quality of reference products and the quality of generated products for (**a**) Steam Community, (**b**) SCP-wiki, and (**c**) Archive of Our Own. The *x*-axis represents the indicator of quality diversity of reference products for each dataset, and the *y*-axis represents the quality of generated products. In the graphs in the left column, the horizontal axis is divided into intervals of fixed width, and the average value of the evaluation for generated products corresponding to each interval is shown by a solid line; the 95% confidence interval for each average value calculated by a two-sided *t*-test is shown by shading (note that there are parts where the shading width is extremely narrow and invisible as well as parts where the confidence interval diverges owing to a small amount of data). The graphs of *d*-degree functions obtained by polynomial regression analysis are shown in the right column. The scale of the *y*-axis is the same as that of the graphs in the left column.

**Figure 2:**
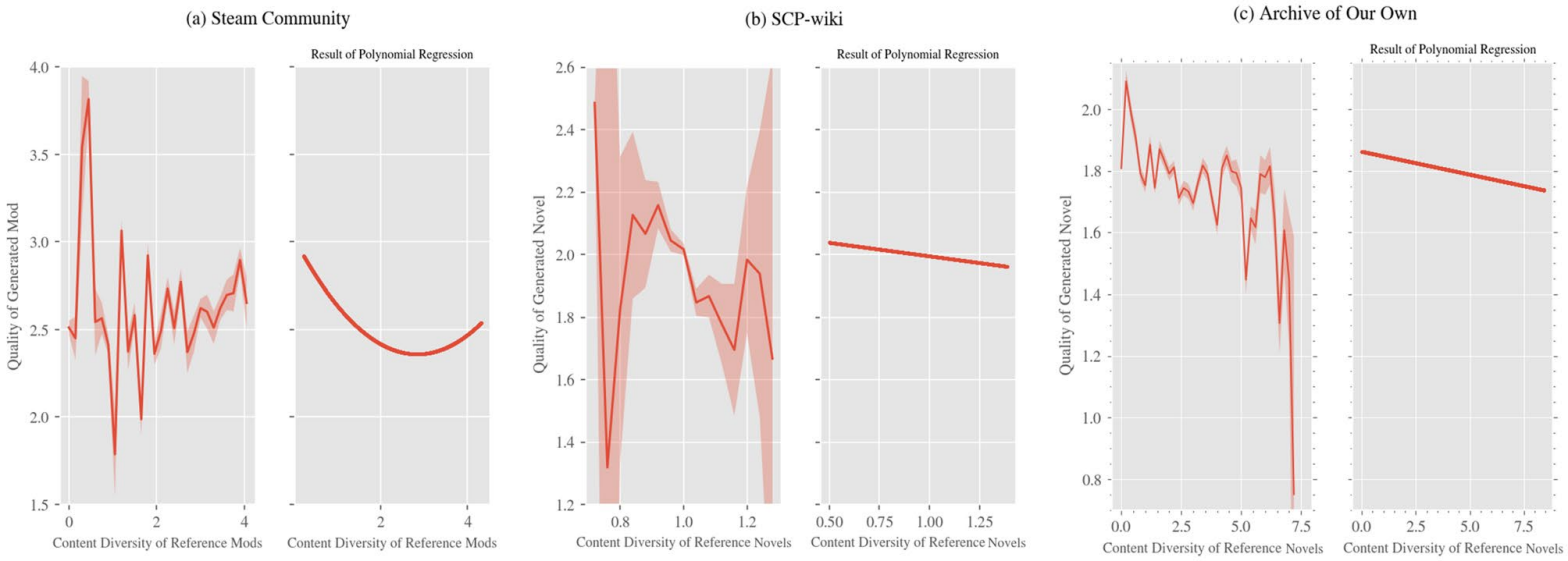
Relationship between the content diversity of reference products and the quality of generated products. This figure shows the relationship between the content diversity of reference products and the quality of generated products for (**a**) Steam Community, (**b**) SCP-wiki, and (**c**) Archive of Our Own. The *x*-axis represents the indicator of content diversity of reference products for each dataset, and the *y*-axis represents the quality of generated products. In the graphs in the left column, the horizontal axis is divided into intervals of fixed width, and the average value of the evaluation for generated products corresponding to each interval is shown by a solid line; the 95% confidence interval for each average value calculated by a two-sided *t*-test is shown by shading (note that there are parts where the shading width is extremely narrow and invisible as well as parts where the confidence interval diverges owing to a small amount of data). The graphs of *d*-degree functions obtained by polynomial regression analysis are shown in the right column. The scale of the *y*-axis is the same as that of the graphs in the left column.

As a result of the regression analysis on the quality diversity of reference products (see Table 1 for the results of the regression analysis, and Fig. 1 for the regression curve), a concave quadratic function was obtained for all three datasets. That is, when the quality diversity of the reference products was neither high nor low, the quality of the generated products was the highest. Meanwhile, as a result of the regression analysis on the content diversity of reference products (see Table 2 for the results of the regression analysis, and Fig. 2 for the regression curve), a convex quadratic function was obtained for Steam Community, and a linear function with a negative coefficient was obtained for SCP-wiki and Archive of Our Own. In the case of Steam Community, when the content diversity indicator was above 2.833, the quality of generated products increased with increasing diversity; however, in other ranges for Steam Community and the other two datasets, there was a tendency for increasing diversity to lower the quality of generated products.

**Table 1:** Results of polynomial regression analysis on the quality diversity of reference products for Steam Community, SCP-wiki, and Archive of Our Own.

Steam Community		SCP-wiki		Archive of Our Own	
Variable	Coefficient	Variable	Coefficient	Variable	Coefficient
*x* (Quality Diversity)	− 6.3421*** (0.887)	*x* (Quality Diversity)	− 1.4312*** (0.350)	*x* (Quality Diversity)	9.9146*** (0.634)
*x*^2^	− 8.9917*** (0.907)	*x*^2^	− 3.5901*** (0.351)	*x*^2^	− 3.7745*** (0.634)
Cities: Skylines Playtime	0.1793*** (0.009)	Number of content revisions	0.0898*** (0.007)	Popularity of the original work referred to	0.1756*** (0.002)
Number of games purchased on Steam	0.0556*** (0.008)	Number of previous participations of the author	0.0025 (0.007)	Order of the target novel among the ones written by its author	− 0.0386*** (0.002)
Order of the mod among the ones developed by its developer	0.3770*** (0.008)	Time point of publication	− 0.1766*** (0.007)		
		Days the author was observed	0.0572*** (0.008)		
Constant	2.6708*** (0.008)	Constant	1.9974*** (0.006)	Constant	1.7817*** (0.002)
R^2^	0.245	R^2^	0.320	R^2^	0.082
BIC	3.543 × 10^4^	BIC	2.060 × 10^3^	BIC	1.249 × 10^5^
Observations	13,623	Observations	2907	Observations	65,619

*p < 0.1, **p < 0.05, ***p < 0.01. Figures in parentheses are standard errors. As all control variables in the table are estimated based on standardized variables, the sizes of the coefficients are comparable.

**Table 2:** Results of polynomial regression analysis on the content diversity of reference products for Steam Community, SCP-wiki, and Archive of Our Own.

Steam Community		SCP-wiki		Archive of Our Own	
Variable	Coefficient	Variable	Coefficient	Variable	Coefficient
*x* (Content Diversity)	− 7.7740*** (0.897)	*x* (Quality Diversity)	− 1.5634*** (.0.364)	*x* (Quality Diversity)	− 2.5591*** (0.675)
*x*^2^	7.2527*** (0.897)				
Cities: Skylines Playtime	0.1723*** (0.009)	Number of content revisions	0.0907*** (0.007)	Popularity of the original work referred to	0.1751*** (0.002)
Number of games purchased on Steam	0.0281*** (0.008)	Number of previous participations of the author	0.0087 (0.007)	Order of the target novel among the ones written by its author	− 0.0233*** (0.002)
Order of the mod among the ones developed by its developer	0.3958*** (0.009)	Time point of publication	− 0.1887*** (0.007)		
		Days the author was observed	0.0769*** (0.008)		
Constant	2.6329*** (0.008)	Constant	1.9884*** (0.006)	Constant	1.7747*** (0.002)
R^2^	0.235	R^2^	0.304	R^2^	0.075
BIC	3.469 × 10^4^	BIC	2.269 × 10^3^	BIC	1.352 × 10^5^
Observations	13,247	Observations	2907	Observations	70,665

*p < 0.1, **p < 0.05, ***p < 0.01. Figures in parentheses are standard errors. As all control variables in the table are estimated based on standardized variables, the sizes of the coefficients are comparable.

### Effects of the quality of reference products

We then examined how the quality of reference products affected the quality of generated products. We defined the quality of reference products as the average quality of all the products contained in the set of reference products. To examine the relationship between the average quality of reference products and the quality of generated products, we performed a polynomial regression analysis. For each dataset, we controlled for the necessary variables (see further details in the subsection “Overview of the Analysis”). The degree of the polynomial was determined based on the BIC.

**Figure 3:**
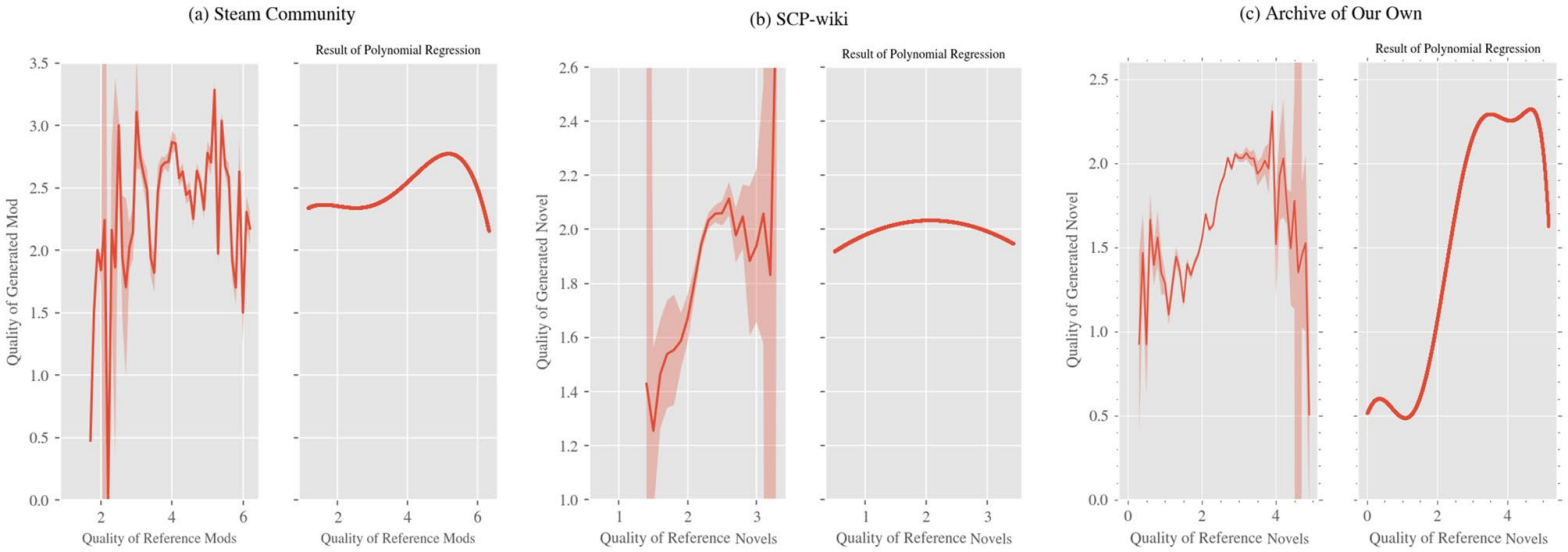
Relationship between the quality of reference products and the quality of generated products. This figure shows the relationship between the average of the quality of reference products and the quality of generated products for (**a**) Steam Community, (**b**) SCP-wiki, and (**c**) Archive of Our Own. The *x*-axis represents the indicator of the average of the quality of reference products for each dataset, and the *y*-axis represents the quality of generated products. In the graphs in the left column, the horizontal axis is divided into intervals of fixed width, and the average value of the evaluation for generated products corresponding to each interval is shown by a solid line; the 95% confidence interval for each average value calculated by a two-sided *t*-test is shown by shading (note that there are parts where the shading width is extremely narrow and invisible as well as parts where the confidence interval diverges owing to a small amount of data). The graphs of *d*-degree functions obtained by polynomial regression analysis are shown in the right column. The scale of the *y*-axis is the same as that of the graphs in the left column.

As a result of the regression analysis on the average quality of reference products (see Table 3 for the results of the regression analysis, and Fig. 3 for the regression curve), we obtained a quartic function for Steam Community, a quadratic function for SCP-wiki, and a seventh-degree function for Archive of Our Own. When the average quality of reference products was above 5.159, 2.534, and 4.680 in Steam Community, SCP-wiki, and Archive of Our Own, respectively, the following tendency was observed: the higher the average quality of reference products, the lower the quality of generated products. This suggests that when only extremely high-quality products are referred to, it is difficult to generate high-quality products.

**Table 3:** Results of polynomial regression analysis on the quality of reference products for Steam Community, SCP-wiki, and Archive of Our Own.

Steam Community		SCP-wiki		Archive of Our Own	
Variable	Coefficient	Variable	Coefficient	Variable	Coefficient
*x* (Average of Quality)	7.5626*** (0.940)	*x* (Average of Quality)	0.5051 (0.396)	*x* (Average of Quality)	48.8276*** (0.623)
*x*^2^	− 2.9418*** (0.939)	*x*^2^	− 2.3781*** (0.356)	*x*^2^	− 8.9341*** (0.599)
*x*^3^	− 7.4377 ***(0.933)			*x*^3^	− 17.8947*** (0.600)
*x*^4^	− 3.7425*** (0.938)			*x*^4^	3.7190*** (0.599)
				*x*^5^	3.7508*** (0.599)
				*x*^6^	− 6.0046*** (0.599)
				*x*^7^	− 2.7173*** (0.599)
Cities: Skylines Playtime	0.2036*** (0.008)	Number of content revisions	0.0910*** (0.007)	Popularity of the original work referred to	0.1231*** (0.002)
Number of games purchased on Steam	0.0778*** (0.008)	Number of previous participations of the author	0.0038 (0.007)	Order of the target novel among the ones written by its author	− 0.0158*** (0.002)
Order of the mod among the ones developed by its developer	0.3224*** (0.008)	Time point of publication	− 0.1848*** (0.008)		
		Days the author was observed	0.0738*** (0.008)		
Constant	2.6112*** (0.007)	Constant	1.9892*** (0.006)	Constant	1.7750*** (0.002)
R^2^	0.189	R^2^	0.309	R^2^	0.164
BIC	4.349 × 10^4^	BIC	2.245 × 10^3^	BIC	1.273 × 10^5^
Observations	16,113	Observations	2964	Observations	70,200

*p < 0.1, **p < 0.05, ***p < 0.01. Figures in parentheses are standard errors. As all control variables in the table are estimated based on standardized variables, the sizes of the coefficients are comparable.

## Discussion

Through the two analyses conducted in this study, the following key results emerged regarding the relationship between others’ reference product characteristics and the quality of generated products: (1) the quality diversity of reference products generated by others has the most positive impact on the quality of generated products when it is neither high nor low, (2) the content diversity of reference products generated by others has a negative impact on the quality of generated products, and (3) the quality of reference products generated by others has a negative impact on the quality of generated products when it is extremely high.

It should be noted that the shapes of the curves obtained from the regression analyses differ slightly among the analyses. This may be due to differences in the diversity metrics of the reference products or differences in the processes and content of creative activities in each community with the dataset we used. The production of creative products involves diverse processes, each of which is influenced by different factors. Therefore, while an analysis reflecting the specific processes of generating ideas and products was difficult with the data used in this study, conducting such an analysis is an important direction for future research.

Consistent with numerous previous studies suggesting the positive effects of diversity on creativity^11,37,62–66^, we hypothesized that the higher the diversity, the higher the quality of the generated products. However, our results surprisingly diverged from this hypothesis, demonstrating an inverted U-shaped relationship between the quality diversity of reference products and the quality of generated products. This finding indicates that excessive diversity negatively affects product generation. This divergence persists with regard to reference products’ content diversity: lower diversity results in a higher quality of generated products. The following reasons could explain why both types of diversity have a negative impact on creativity.

Ideation is an important process for producing creative works^74^. Many studies have shown that memory exploration is important in ideation^75,76^. The extent of memory exploration is, however, suggested to decrease owing to the cognitive load^77^ from exposure to excessively diverse information^78^. Therefore, when exposed to an excessively diverse set of ideas/products from other people, the amount of internal exploration that is important for ideation may be suppressed, which may have a negative impact on generating high-quality ideas. In addition, the awareness of differentiation from existing products in order to generate original products may negatively affect the quality of the products. In other words, by being exposed to other people’s diverse products, some people may feel more constrained about the content of products that can be posted to the community, which may reduce creative performance^52^. However, the above discussion is speculative; examining the potential relationship between the cognitive mechanisms of creativity and the aforementioned empirical results is beyond the scope of this research. We believe that this topic could be a promising direction for future research to foster more in-depth discussions.

Alternatively, the following interpretation of the diversity of other people’s products is possible. Other people’s products considered in this study (which we referred to as reference products) were limited to works belonging to the same genre as the work that one was trying to create. Thus, the diversity of other people’s products is limited. However, previous studies have shown that effective diversity for creativity (e.g., broad knowledge^11^ or word diversity encountered during idea generation^37^) constitutes all information diversity that extends beyond one’s own creative domain. Therefore, we can consider that diverse knowledge about other people’s products does not necessarily imply sufficiently diverse information necessary for high creativity, but rather relates to diversity that exists in a narrow range of scope.

Regarding the quality of reference products, we hypothesized that a higher quality of reference products would lead to a higher quality of generated products. However, our analysis revealed an inverted U-shaped relationship in this regard. That is, when the quality of reference products is too high, it may have a negative impact on the quality of generated products.

One possible reason for this result may be related to fixation, a phenomenon of adherence where people tend to generate similar ideas based on inherited features from example ideas presented in creative generation tasks^57–60^. A possible explanation for why high-quality ideas cause fixation is as follows. High-quality ideas/products are more memorable and more frequently recalled. Even in the ideation process where one expands their own idea, that idea/product is frequently recalled, and one’s thinking does not move away from it when the idea/product is of high quality. Consequently, the generation of novel ideas is inhibited, and inferior versions of high-quality example ideas are generated. However, this hypothesis could not be tested in this study and thus requires future analysis.

Of course, this does not necessarily mean that referring to high-quality products will always result in the inability to generate high-quality products. For example, professional creators who have produced masterpieces are unlikely to be unaware of other masterpieces within the genre they work in. Although high-quality products should provide creators with hints on how to improve the quality of their own products, the results of this study suggest that many people tend to be unable to leverage them effectively. This suggests that merely referring to other people’s products, while necessary, is not sufficient for generating high-quality products. Specifying the further conditions necessary to leverage high-quality products in the creation of one's own products is an important direction for future research.

In summary, this study demonstrates that referring to other people’s products that are not too diverse and not too high in quality may facilitate the generation of high-quality products.

We conclude by highlighting the limitations of this study. First, the results regarding the relationship between the characteristics of reference products and quality of generated products do not necessarily indicate a causal relationship. Therefore, other interpretations of this relationship than causality are possible. For example, people who are especially talented in a domain may have unique inclinations toward referring to others’ works, even if those works do not influence the quality of their outputs. Second, for methodological tractability, we employed “Favorite,” “Vote,” and “Bookmark” records to identify the referring relationship between products. However, the reliability of this metric could vary across users. That is, some users may record all the products they viewed instead of the products they genuinely preferred, or some may record others’ products as material for differentiating their own products. Additionally, it should be noted that with the metrics used in this study, we could not accurately capture the extent to which the referenced products influenced one's own products. This is because not all referenced products contribute equally to idea generation. Third, the information affecting idea generation does not exist solely within the online community in which products are posted. From the three datasets used in this study, we were unable to capture creator’s activities in a diverse information space that extends beyond the community. To this end, examining web browsing history data could be an option for further research. Fourth, the creative activities examined in this study were limited to mod development and novel writing. It cannot be ruled out that the effects of reference products may differ for other forms of creative activity. Therefore, further research is required to validate our results in the context of other creative activities. Fifth, we were unable to investigate the cognitive mechanisms underlying the complex relationship between the characteristics of reference products and the quality of generated products. To elucidate the cognitive mechanisms that use reference products as material for idea/product generation in creative activities, further exploration is necessary through data analysis such as that employed in this study, as well as through psychological experiments.

Additionally, a promising direction for future research to deepen our understanding of this topic is investigating whether fixation occurs when referring to high-quality products. When conducting analyses using methods such as those employed in this study (utilizing large-scale datasets), creative works with text-based formats, such as novels, can be focused on. By employing natural language processing to project text into vectors, various analysis methods can be considered. For instance, future research could explore whether the text vectors in novels written after referring to products are closer to reference product vectors, which can be regarded as fixation, as well as the relationship between the strength of fixation and the quality of the reference products. In addition, analyzing the content diversity of generated products, which reflects one aspect of creativity, is also important. While it appears that the diversity of reference products has a negative impact on the quality of generated products, there is a possibility that the content diversity of the generated products has a positive impact. Furthermore, by utilizing novel text data for analysis, new methods for measuring the quality of works from narratives^79^ can also be employed. Moreover, creativity is influenced by many factors beyond just the quality and diversity of reference products. In this study, we were unable to sufficiently analyze important factors in creativity within online communities, such as enhancing work quality through careful examination with an audience in mind for content release^46,80^, as well as communication and relationships with other users in the community^81^ (for example, in the community analyzed in this study, comments on works, discussions, friendships within the community). Analyzing the above matters using data that can be obtained from communities is an important direction for future research. We hope that the findings of this study regarding the relationship between referring to products and creativity will be used as reference material, or “reference products,” to gain new insights into creativity.

## Materials and methods

### Description of the three datasets

In this study, we collected data from Steam Community for Cities: Skylines mod development community (https://steamcommunity.com/app/255710/workshop/), SCP-wiki (http://www.scp-wiki.net), and Archive of Our Own (https://archiveofourown.org/), and examined the effect of referring to existing products on the generation of novel products.

Steam Community is a community within Steam, the world’s largest computer game platform, where game players can post and interact with the works they create. In this study, we collected data from the Cities: Skylines community, where mod development is most active. From Steam Community, we used web scraping with a Python program to collect 170,032 mods developed by 35,579 developers by September 13, 2022. For each mod, the information obtained from the community was as follows: information on the developer (discussed below); current subscribers (the number of players currently using the mod in the game); publication date; and tags (122 types of tags that succinctly represent the content of the mod). In addition, the information collected for each developer was as follows: Cities: Skylines playtime (this information can be personalized by the players themselves; thus, only the data of 10,665 people were collected); the number of games purchased on Steam (this information can also be personalized by the players themselves; thus, only data of 11,754 people were collected); and the list of Cities: Skylines mods that they added to their favorites.

From SCP-wiki, using the API provided, we collected 4653 SCP-stories (novels) posted by 3405 participants from January 2009 to December 2018. The information collected for each novel was as follows: who voted for that novel, rating (value of (+ 1 votes)−(− 1 votes)), initial draft text, and publication date.

From Archive of Our Own, we collected 102,964 novels written by 6031 randomly selected authors using web scraping with a Python program. The information collected for each novel was as follows: kudos rate (the number of times the readers selected the heart icon for the novel), original work status, authorship, and publication date. In addition, we collected information on 573,012 novels bookmarked by the authors, including the date and time of bookmarking, kudos rate, original work status, authorship, and publication date.

Note that data collection was performed within the limits of the API when using it, and when using a Python program, the page was accessed at the same speed as a human would manually browse the community, taking great care not to overload the website. In addition, no personal information or the content of the work itself (except for SCP-wiki) was collected.

### Metrics

1. Quality of Products

To investigate the effect of referring to other people’s products on the quality of creative products, we set quality indicators of creative products for each of the three communities.

For Steam Community, we used the value of “Current Subscribers” to measure the quality of mods. This value represents the number of people currently using mods in the game. Cities: Skylines mods fall into two categories: those that emphasize functionality and those that emphasize design (the latter are called “Assets”). Mods with a large value of “Current Subscribers,” that is, mods with a large number of users, can be said to be high-quality mods, as either their functionality or design is highly rated by players.

For SCP-wiki, we used the value of “Rating” to measure the quality of novels. Participants could vote “ + 1” or “− 1” for each novel, where “ + 1” indicates that the content of the novel is good, whereas “− 1” indicates that it is bad. The rating was the sum of all participants’ “ + 1” and “− 1” votes, reflecting the quality of the novel.

For Archive of Our Own, we used the value of “Kudos” to measure the quality of novels. On this platform, readers can leave hearts for novels they think have good content. The total number of hearts thus left for a novel represents the kudos rate, which reflects the quality of the novel.

The final indicators of creative product quality were obtained by taking the base 10 logarithm for each community as follows:

$$\text{Quality of Steam Community Mod}={\log}_{10}(1+\text{Current Subscribers})$$

$$\text{Quality of SCP-wiki novel}=\log_{10}(1+\text{Rating})$$

$$\text{Quality of Archive of Our Own novel}=\log_{10}(1+\text{Kudos})$$

We used a logarithmic scale because the distributions of Current Subscribers, Rating, and Kudos were right-skewed, or long-tailed in the direction of large values.

(2) Sets of Reference Products

We determined the sets of other people’s products that the creators referred to during product generation using the referring behavior data recorded in each community. For Steam Community, we regarded adding a product to the “Favorite” list as a referring behavior, as it indicates preference for the product. For SCP-wiki, we regarded voting for a novel as a referring behavior because it implies reading the novel carefully. For Archive of Our Own, we regarded adding a “Bookmark” as a referring behavior, as it indicates preference for the novel. As the reference product set for each mod or novel, we collected all the products of other people that were judged to be referred to before the development or writing of that mod or novel.

(3) Diversity of Reference Products

To test the hypothesis regarding the diversity of reference products, we defined indicators of reference products’ quality and content diversity. The quality diversity of reference products was defined as the standard deviation of the quality evaluation indicators of all products contained in the set of reference products. The content diversity of reference products was defined differently for each dataset. For Steam Community, each mod has a “tag” that succinctly represents its content. When a set of reference products consisting of *N* products contained *n*_*i*_ mods with a tag *i*, we defined the content diversity of this set of reference products as follows:

$$-\sum_{i}{p}_{i}\log_{2}{p}_{i}$$

where *p*_*i*_ = *n*_*i*_/*N*. We adopted the same definition for Archive of Our Own. When a set of reference products consisting of *N* products contained *n*_*i*_ novels derived from the original product *i*, we defined *p*_*i*_ = *n*_*i*_/*N* and used the same formula as above for this set of reference products’ content diversity. This metric is equivalent to the average information content or Shannon’s entropy, and it can be used to evaluate the diversity of a group (e.g., diversity of cited references in a journal^82,83^, diversity of a biological community^84^, pedodiversity^85^).

The indicator of reference products’ content diversity for SCP-wiki was quite different from the above two; it was defined using the text data of the stories to reflect the detailed content of the products. First, we used a pretrained sentence-embedding model based on BERT^72,73^, a powerful natural language model, to project sentences of stories into 768-dimensional vectors. For each reference product *i* contained in the set of reference products, we denoted the corresponding vector as *v*_*i*_ (*i* ∈ (1, 2, …, *N*)). We then defined the content diversity of this set of reference products as follows:

$$\frac{2}{N\left(N-1\right)}{\sum }_{i<j}{\left|\left|{v}_{i}-{v}_{j}\right|\right|}_{2}$$

where $\left\| \bullet \right\|_{2}$ denotes the 2-norm. That is, we regarded the average distance between all *N*(*N *− 1)/2 pairs of vectors of reference products contained in the set of reference products as diverse.

(4) Quality of Reference Products

To test the hypothesis regarding the quality of reference products, we defined the set of reference products’ quality indicator as the average of the quality indicators of all products contained in the set of reference products.

### Polynomial regression model

To analyze the relationship between reference products’ characteristics and the quality of generated products, we performed a polynomial regression analysis with variables related to reference products’ characteristics as explanatory variables and those related to the quality of generated products as dependent variables. However, to solve the problem of multicollinearity between the terms appearing in the polynomial model, we used orthogonal polynomials obtained by QR decomposition. In addition, we applied L_1_ regularization to prevent overfitting. The degree *d* of the polynomial was determined by the smallest *d* that minimizes the BIC. The regression formulas are as follows:

For Steam Community data:

$$\begin{aligned}{}&\text{Current Subscribers on logarithmic scale}\\&={\beta }_{0}+\sum_{i=1}^{d}{\beta }_{i}{x}^{i} \\ &+\alpha_1\log_{10}\text{Order of the mod among the ones developed by its developer.}\\&+\alpha_2\log_{10}\text{Playtime of Cities: Skylines by the developer.}\\&+\alpha_3\log_{10}\text{Number of games purchased on Steam by the developer.}\end{aligned}$$

For SCP-wiki data:

$$\begin{aligned}{}&\text{Rating on logarithmic scale} \\&= {\beta }_{0}+\sum_{i=1}^{d}{\beta }_{i}{x}^{i} \\ &+ \alpha_1\log_{10}\text{Number of content revisions.} \\ &+\alpha_2\text{Time point of publication.}\\&+\alpha_3\text{Number of days since the author had published their first novel.}\\&+\alpha_4\text{Number of times the author had previously participated.}\end{aligned}$$

For Archive of Our Own data:

$$\begin{aligned}{}&\text{Kudos on logarithmic scale}\\&={\beta }_{0}+\sum_{i=1}^{d}{\beta }_{i}{x}^{i}\\&+ \alpha_1\log_{10}\text{Popularity of the original work referred to.}\\&+\alpha_2\log_{10}\text{Order of the target novel among the ones written by its author.}\end{aligned}$$

### Supplementary Information

Supplementary Information.

## Data Availability

Data and code related to this paper are available at: 10.17605/OSF.IO/S5MZ4.
