# Supplementary material for: Impact of the quality and diversity of reference products on creative activities in online communities
Source: Sci Rep. 2024 Jul 11;14:15316. doi: 10.1038/s41598-024-65124-y (PMC11239815; doi:10.1038/s41598-024-65124-y)
Supplement: Supplementary file 1 — Supplementary Information. [file 41598_2024_65124_MOESM1_ESM.pdf]

Supplementary Material for

Impact of the Quality and Diversity of Reference Products on Creative Activities in Online Communities

Keisuke Sato[1,*], Kunhao Yang[2], Kazuhiro Ueda[1,*]

[1]Graduate School of Arts and Sciences, The University of Tokyo, Tokyo, Japan (postal code 153-8902)

[2]Graduate School of Sciences and Technology for Innovation, Yamaguchi University, Yamaguchi, Japan (postal code 755-8611)

Email: lesucre326@g.ecc.u-tokyo.ac.jp, ueda@g.ecc.u-tokyo.ac.jp

This PDF file includes:

Supporting text
Figures S1 to S3
Tables S1 to S2

S1. Statistical information regarding the variables used in the polynomial regression models for Steam Community, SCP-wiki, and Archive of Our Own

In the manuscript, polynomial regression models were used to examine the relationship between the characteristics of reference products and of generated products. The statistical information regarding the dependent variable, the independent variable, and the control variables in each regression model are shown in Table S1 and the correlations between these variables are shown in Figures S1-S3.

Fig. S1. The correlations among the variables in the regression model for Steam Community reported in the manuscript. The correlation coefficients are shown in a matrix with variable names in the diagonal. The color and number of every element in the matrix shows the correlation coefficient values.

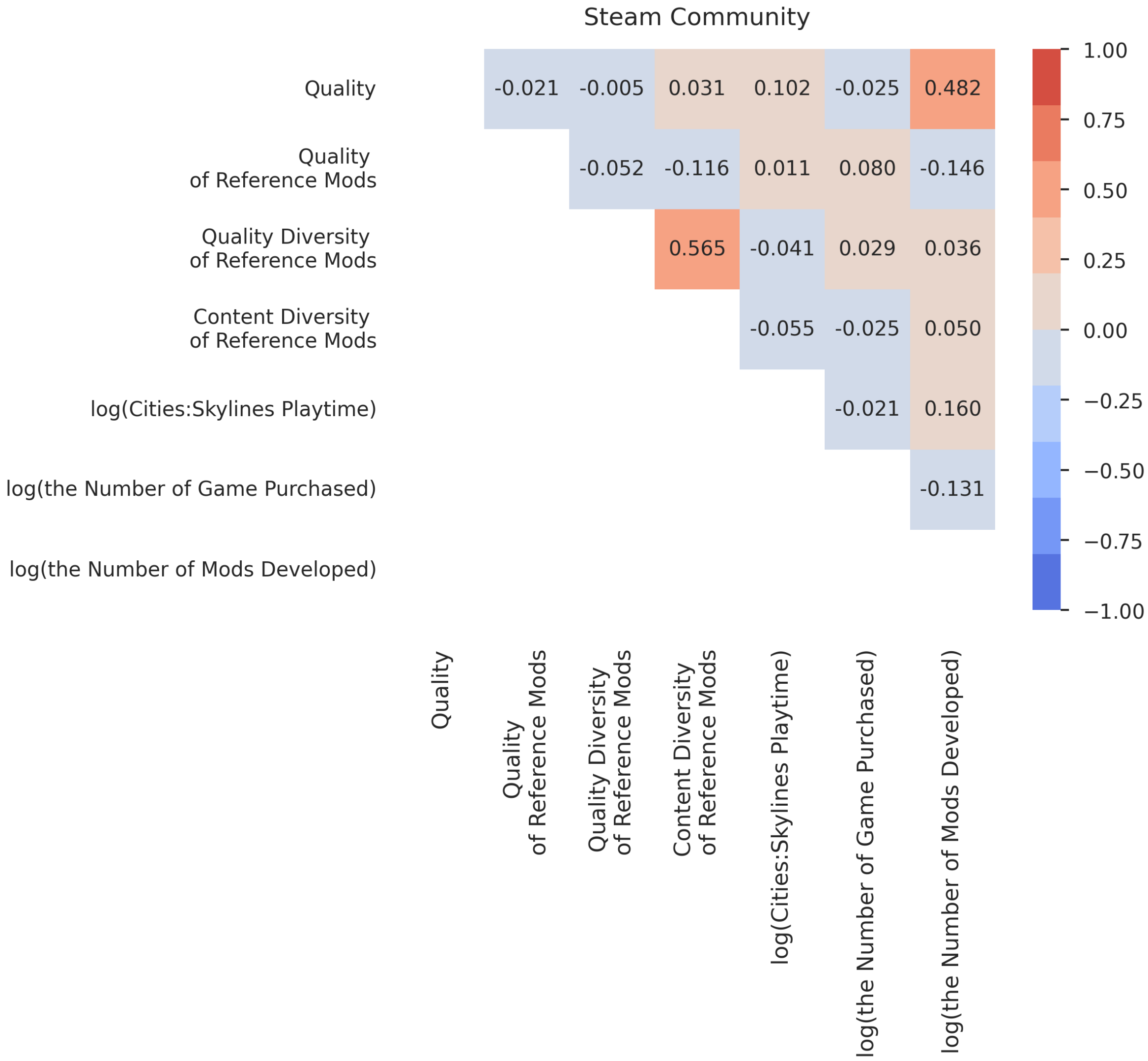

Fig. S2. The correlations among the variables in the regression model for SCP-wiki reported in the manuscript. The correlation coefficients are shown in a matrix with the variable names in the diagonal. The color and number of every element in the matrix shows the correlation coefficient values.

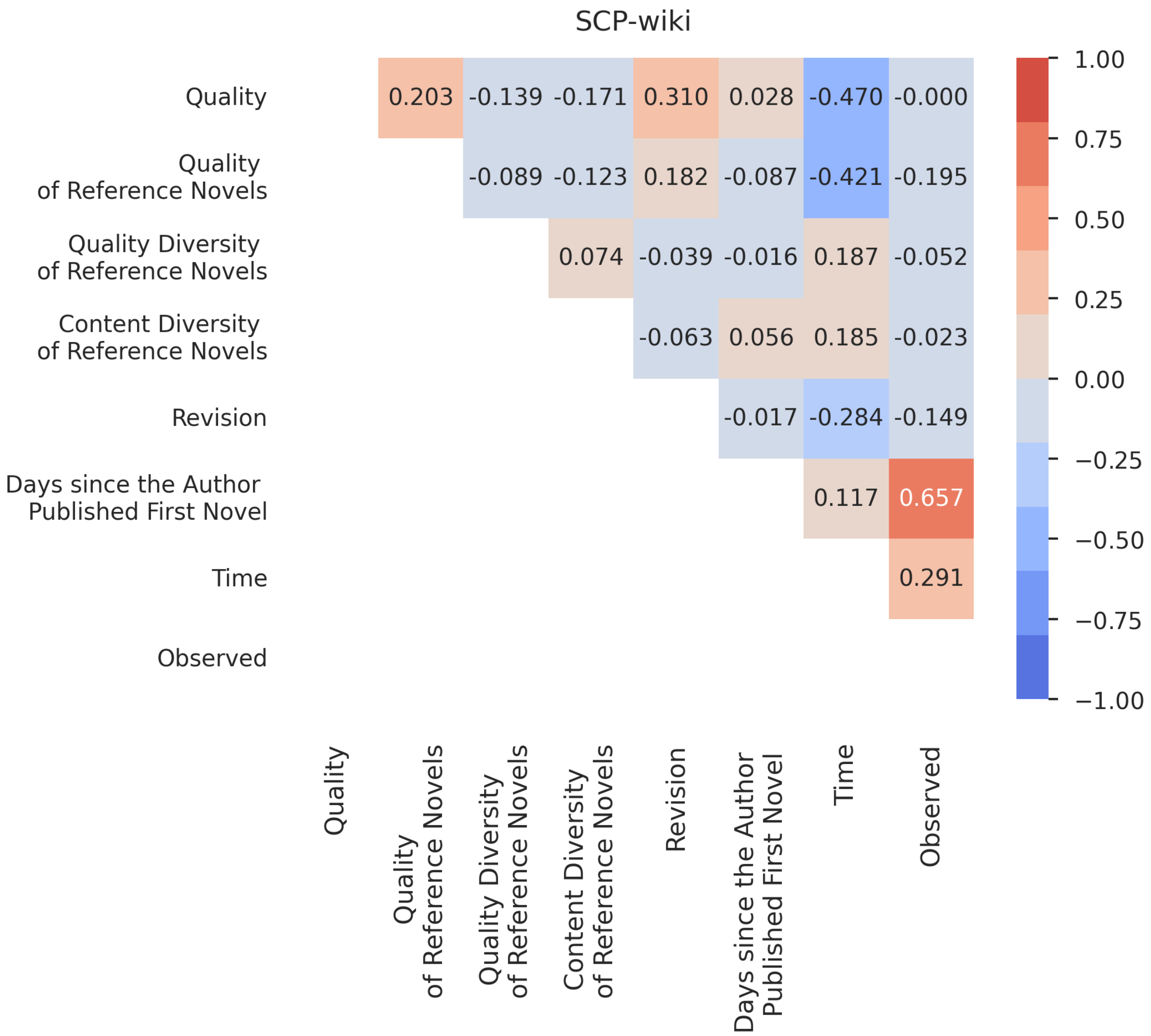

Fig. S3. The correlations among the variables in the regression model for Archive of Our Own reported in the manuscript. The correlation coefficients are shown in a matrix with the variable names in the diagonal. The color and number of every element in the matrix shows the correlation coefficient values.

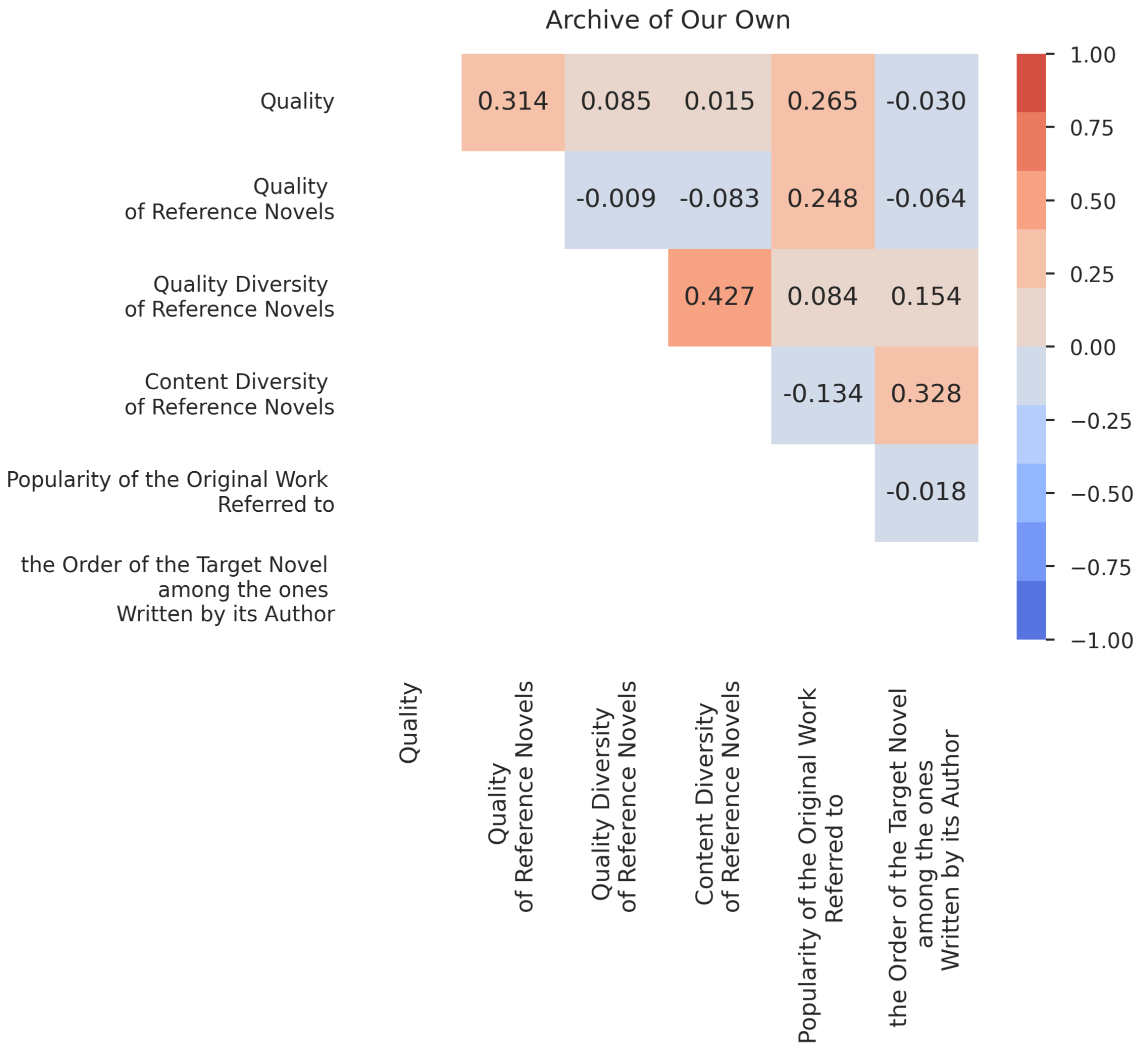

Table S1. Statistical Information for variables in the Steam Community, SCP-wiki, Archive of Our Own datasets

Steam Community					
**Name**	**Count**	**Mean**	**Std. Dev.**	**Min**	**Max**
Average of the quality of all mods developed by the developer	35,579	1.720	0.824	0.000	6.300
Current Subscribers on logarithmic scale (Quality of Mods)	170,035	2.225	1.137	0.000	6.315
Quality of reference mods	95,544	4.379	0.785	0.301	6.315
Quality diversity of reference mods	95,544	0.495	0.389	0.000	2.751
Content Diversity of reference mods	95,518	14.375	14.728	1	65
The number of mods developed by each developer	35,579	4.780	19.952	1	1191
Playtime of Cities: Skylines by the developer	10,665	4.100	0.544	1.591	6.116
The number of games purchased on Steam by the developer	11,754	2.121	0.516	0.301	4.315
**SCP-wiki**					
**Name**	**Count**	**Mean**	**Std. Dev.**	**Min**	**Max**
Rating on logarithmic scale (Quality of novels)	4,848	2.007	0.409	0.000	3.716
Quality of reference novels	3,068	2.395	0.235	0.477	3.716
Quality diversity of reference novels	2,984	0.406	0.093	0.015	0.858
Content diversity of reference novels	4,434	1.018	0.042	0.757	1.387
The number of content revisions	4,848	13.178	12.237	0	261
The number of times the author had previously participated	4,845	171.964	427.680	0	3,736
*The point of publication	4,845	0.000	1.000	-1.973	1.355
The number of days since the author had published his/her first novel	4,845	524.774	777.555	0	3,923
**Archive of Our Own**					
**Name**	**Count**	**Mean**	**Std. Dev.**	**Min**	**Max**
Kudos on logarithmic scale (Quality of novels)	102,964	1.734	0.659	0.000	4.416

Quality of reference novels	80,850	2.471	0.562	0.000	5.164
Quality diversity of Reference novels	80,850	0.690	0.367	0.000	2.291
Content diversity of Reference novels	102,964	1.965	1.801	0.000	8.439
Popularity of the original work referred to	102,964	59.327	95.832	1	776
The order of the target novel among the ones written by its author	88,299	4.126	0.938	0.301	5.774

* “The point of publication” is a standardization of date data

S2. Bayesian information criterion values in polynomial regression analysis for Steam Community, SCP-wiki, and Archive of Our Own

In the manuscript, polynomial regression models were used to examine the relationship between the characteristics of reference products and of generated products. The degree of the polynomial was determined by Bayesian information criterion. The values of the Bayesian information criterion for polynomial regression models for various degrees are listed in Table S2.

Table S2. Bayesian information criterion values for each degree in the polynomial regression model

Steam Community			
**Independent Variables**	**Quality of Reference Products**	**Quality Diversity of Reference Products**	**Content Diversity of Reference Products**
**Degree**	**BIC**	**BIC**	**BIC**
1	43554.482	35514.941	34746.949
2	43554.112	35426.489	34691.215
3	43500.595	35431.331	34700.642
4	43494.368	35437.610	34642.190
5	43498.100	35406.019	34637.584
6	43505.820	35408.589	34526.795
7	43509.691	35395.718	34536.149
8	43443.996	35405.214	34463.475
9	43448.572	35335.242	34429.923
10	43362.636	35293.041	34415.403
**SCP-wiki**			
**Independent Variables**	**Quality of Reference Products**	**Quality Diversity of Reference Products**	**Content Diversity of Reference Products**
**Degree**	**BIC**	**BIC**	**BIC**
1	2284.912	2155.191	2268.594
2	2257.437	2060.219	2269.841
3	2261.714	2068.112	2245.926
4	2254.260	2062.334	2253.015
5	2260.570	2066.366	2253.329
6	2266.267	2071.639	2258.903
7	2270.473	2073.856	2258.607
8	2278.392	2081.786	2266.388
9	2286.244	2088.769	2263.765
10	2292.676	2095.837	2271.623
**Archive of Our Own**			
**Independent Variables**	**Quality of Reference Products**	**Quality Diversity of Reference Products**	**Content Diversity of Reference Products**
**Degree**	**BIC**	**BIC**	**BIC**
1	128571.667	124882.654	135156.781
2	128366.035	124858.306	135162.736
3	127496.984	124859.369	135141.899
4	127469.730	124869.930	135095.942
5	127441.793	124878.121	135106.140
6	127352.515	124868.312	135109.950
7	127343.103	124873.820	135101.265
8	127353.507	124840.211	135111.113
9	127275.541	124850.435	134964.365

10	127282.179	124859.222	134963.958

Red letters refer to the selected degree.
